# Gut microbiota-mediated chemotherapy resistance in colorectal cancer: mechanisms and precision interventions

**DOI:** 10.3389/fonc.2026.1966862

**Published:** 2026-09-14

**Authors:** Yan Li, Fangrui Wang, Tao Li, Ying Liu

**Affiliations:** 1 School of Medicine, Medical College, Wuhan University of Science and Technology, Wuhan, Hubei, China; 2 Department of Laboratory Medicine, General Hospital of Central Theater Command, Wuhan, Hubei, China; 3 Hubei Key Laboratory of Central Nervous System Tumors and Intervention, Wuhan, Hubei, China

**Keywords:** drug resistance, neoplasm, colorectal cancer, gut microbiota, microbiome-based intervention, tumor microenvironment

## Abstract

Colorectal cancer (CRC) is a major global health burden, and chemotherapy resistance remains a major challenge to effective treatment. Increasing evidence indicates that the gut microbiota may contribute to variability in chemotherapy response in CRC through microbial metabolism, tumor-cell adaptation, immune regulation, and microbiota-derived metabolites. These processes can alter drug exposure, cellular stress responses, antitumor immunity, and metabolic conditions within the tumor–host ecosystem. In this review, we summarize current evidence on the mechanisms linking the gut microbiota to chemotherapy response in CRC, focusing on microbial drug metabolism, tumor-cell adaptation, immune and metabolic regulation, and microbial interactions with host signaling pathways. We also discuss emerging microbiome-based interventions, including probiotics, fecal microbiota transplantation, phage therapy, and targeted modulation of microbial functions, while considering the limitations of current evidence and challenges to clinical translation. Finally, we highlight future priorities, including causal validation of microbial functions, identification of robust biomarkers, and prospective evaluation of microbiome-informed patient stratification. This review provides an overview of current mechanistic evidence and the potential of microbiome-based approaches to improve chemotherapy response in CRC.

## 1 Introduction

Colorectal cancer (CRC) is one of the most common malignancies worldwide (1, 2). Chemotherapy remains an important component of CRC treatment, with established roles in adjuvant therapy, neoadjuvant treatment of locally advanced rectal cancer, and systemic treatment of metastatic disease (3). However, substantial interindividual variability exists in chemotherapy sensitivity (4, 5), and both primary and acquired resistance can lead to treatment failure, disease progression, and poor clinical outcomes (6).

The gut microbiota, a key component of the host microbial ecosystem, is increasingly recognized as an important determinant of therapeutic response in CRC (7). Beyond its involvement in CRC development and progression, the gut microbiota can modulate chemotherapy efficacy by influencing the tumor microenvironment, host immunity, and drug metabolism (8–10). However, current evidence supporting these mechanisms is derived mainly from mechanistic and preclinical studies, while clinical evidence remains relatively limited (11). With advances in multi-omics and functional studies, research has increasingly shifted from characterizing differences in microbial composition toward defining microbial functions (12, 13). Microbial functional features, microbiota-derived metabolites, and their interactions with host signaling networks are increasingly recognized as critical links between the gut microbial state and chemotherapy sensitivity (14).

Despite accumulating evidence implicating multiple microbial taxa in the development of chemotherapy resistance in CRC, substantial limitations remain in the current evidence base. Existing studies have relied predominantly on *in vitro* experiments, animal models, and clinical association analyses, and the causal relationships among resistance-associated microbial functional features, key metabolites, and host signaling networks that mediate chemotherapy resistance remain insufficiently established (15). Meanwhile, microbiome-based interventions remain largely at the preclinical stage, and consistent evidence is lacking regarding their efficacy in improving chemotherapy sensitivity and clinical outcomes, the patient populations most likely to benefit, and their long-term safety.

Against this background, this review focuses on the key mechanisms underlying gut microbiota-mediated chemotherapy resistance in CRC, with particular emphasis on microbial regulation of chemotherapy drug metabolism, tumor cell adaptation, immune microenvironment remodeling, and microbiota-derived metabolite-mediated therapeutic resistance. We further summarize current microbiome-based intervention strategies and advances in clinical translation, thereby providing a framework for understanding the mechanisms of chemotherapy resistance and developing precision microbiome-based interventions.

## 2 Remodeling of the gut microbiome under chemotherapy pressure

Early studies primarily focused on differences in microbial composition between patients with CRC and healthy individuals. More recent research has recognized that determinants of chemotherapy sensitivity extend beyond microbial composition to functional features encoded by the microbiota, including drug-metabolizing enzymes, virulence factors, and microbial metabolites (16–18).

Chemotherapeutic agents exert antitumor effects while simultaneously disrupting intestinal microbial homeostasis (19). Commonly used regimens containing 5-fluorouracil (5-FU) and oxaliplatin can induce intestinal mucosal injury, exacerbate inflammation, and impair intestinal barrier function, accompanied by alterations in microbial diversity and community composition, including depletion of some commensal bacteria (20–22). Longitudinal studies further indicate that chemotherapy is associated with temporal remodeling of the gut microbiome, with changes in the relative abundance of individual taxa and overall community structure observed at different stages of treatment (23, 24). These dynamic changes may in turn influence microbial functions and host–microbiota interactions, providing an important context for investigating how gut microbiota-associated mechanisms may contribute to chemotherapy resistance in CRC.

## 3 Mechanisms underlying gut microbiota-mediated chemotherapy resistance in CRC

Current evidence indicates that the gut microbiota can influence the development and persistence of chemotherapy resistance through multiple interconnected biological processes, including drug metabolism, cellular damage responses, tumor cell phenotypic plasticity, DNA damage responses, immune microenvironment remodeling, and microbiota-derived metabolite signaling (25). Representative resistance-associated microbial taxa, key effector molecules, and their principal mechanisms of action are summarized in **Table 1**, with a focus on representative examples supported by relatively substantial evidence to date. The major mechanisms are illustrated in **Figure 1**.

**Table 1:** Representative gut microbial taxa influencing chemotherapy response in CRC and their mechanisms of action.

Representative gut microbial taxa	Key effector molecule	Major mechanism	Associated chemotherapeutic agents	Impact on chemotherapy response
*Fusobacterium nucleatum (*26, 27)	Lipopolysaccharide (LPS); miR-18a*; miR-4802	Activates the TLR4/MyD88/NF-κB signaling pathway, promotes protective autophagy, and modulates programmed cell death	5-FU, OXA	Promotes chemotherapy resistance and is associated with poorer treatment outcomes
Enterotoxigenic *Bacteroides fragilis* (ETBF) (28)	SusD/RagB; *B. fragilis* toxin (BFT)	SusD/RagB activates NOTCH1 signaling to promote EMT and cancer stemness; BFT disrupts epithelial integrity and activates inflammatory signaling	5-FU, OXA	Promotes resistance to 5-FU and oxaliplatin through NOTCH1-dependent EMT and cancer stemness; phage-mediated depletion of ETBF restores chemosensitivity
*pks^+^ Escherichia coli* (29, 30)	Colibactin	Induces DNA damage and alters the DNA damage response	OXA, irinotecan	May impair treatment response by promoting DNA damage tolerance and tumor adaptation
Bacteria harboring the *preTA* operon (31)	*PreTA*-encoded enzymes	Metabolize 5-FU, thereby reducing effective drug exposure	5-FU	Reduces effective drug exposure and may attenuate chemotherapy efficacy
β-Glucuronidase (GUS)-producing bacteria (32, 33)	GUS	Catalyzes the deglucuronidation of SN-38G to regenerate SN-38	irinotecan	Primarily contributes to irinotecan-associated toxicity and treatment tolerance; evidence supporting a direct role in chemotherapy resistance remains limited
*Bacteroides vulgatus (*34)	Microbial nucleotide biosynthesis	Promotes microbial nucleotide synthesis and may influence tumor nucleotide metabolism	5-FU-based neoadjuvant chemoradiotherapy	Associated with reduced treatment response

**Figure 1:**
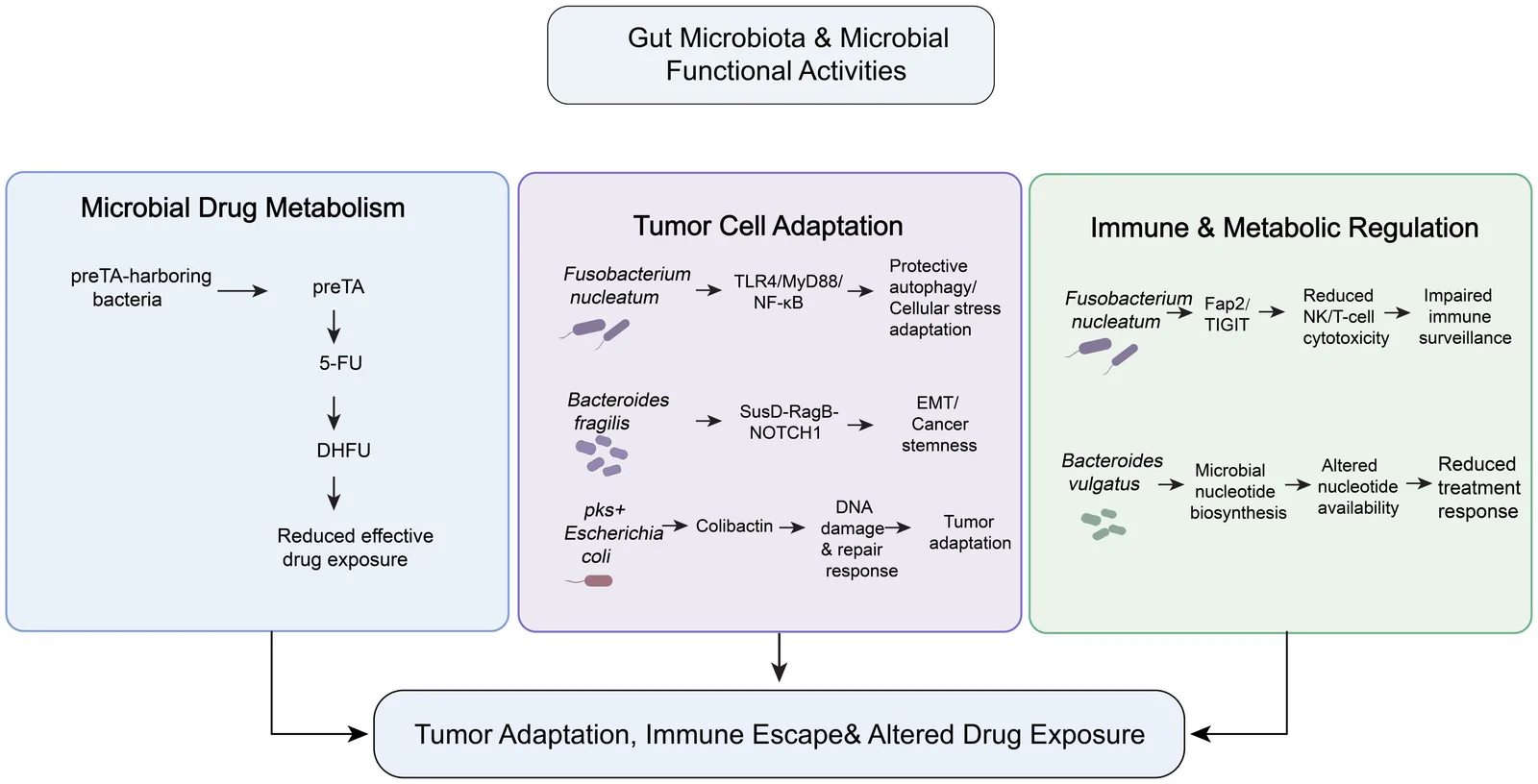
Schematic overview of the major mechanisms by which the gut microbiota influences chemotherapy response in colorectal cancer.

### 3.1 Direct modulation of chemotherapy drug metabolism and effective drug exposure

The emergence of pharmacomicrobiomics has expanded the conventional framework of drug metabolism, highlighting that the effective exposure of chemotherapeutic agents is not determined solely by host metabolic pathways. The gut microbiota can participate in drug biotransformation through the production of specific functional enzymes, thereby altering the active forms and systemic exposure of chemotherapeutic agents (35).

Traditionally, 5-FU was thought to be predominantly inactivated by host dihydropyrimidine dehydrogenase (DPD), while genetic variation in DPYD can influence drug exposure and treatment-related toxicity (36). However, Spanogiannopoulos et al. (31) demonstrated that some gut bacteria harbor the *preTA* operon, which encodes enzymes with DPD-like activity that convert 5-FU into the inactive metabolite dihydrofluorouracil (DHFU). Animal studies further showed that *Escherichia coli* carrying *preTA* can reduce systemic exposure to fluoropyrimidines and attenuate their antitumor activity, providing evidence that microbiota-mediated drug inactivation may complement host drug metabolism. However, the clinical relevance of microbial *preTA* relative to host DPD remains unclear. Although *preTA* has been detected in patients with CRC and has been reported to change during fluoropyrimidine treatment, clinical evidence is currently insufficient to determine the relative contributions of microbial *preTA* and host DPD to 5-FU metabolism (20, 31).

Beyond drug inactivation, certain gut bacteria can also alter local drug exposure through drug reactivation (37). The active irinotecan metabolite SN-38 is glucuronidated by host UDP-glucuronosyltransferase 1A1 (UGT1A1) to form the inactive metabolite SN-38G, which is subsequently delivered to the intestinal lumen. Bacterial β-glucuronidase (GUS) can deconjugate SN-38G back to SN-38, resulting in local accumulation of the active metabolite and intestinal mucosal injury (33). Selective inhibition of bacterial GUS activity can reduce irinotecan-associated intestinal toxicity while preserving its antitumor efficacy (38, 39). However, current evidence concerning GUS is primarily focused on the regulation of irinotecan toxicity, and whether GUS directly contributes to chemotherapy resistance in CRC remains insufficiently established.

Collectively, microbiota-mediated alterations in drug metabolism can influence the effective exposure of chemotherapeutic agents and may modify the selective pressures imposed by chemotherapy. These effects could contribute to the persistence or enrichment of resistance-associated microbial functions and, in turn, influence tumor adaptation to sustained therapeutic pressure.

### 3.2 Microbiota-driven remodeling of tumor cell adaptation and development of chemotherapy resistance

#### 3.2.1 Microbiota-mediated remodeling of cellular damage responses and stress adaptation

The gut microbiota can influence tumor cell survival and chemotherapy sensitivity by reshaping cellular responses to chemotherapy-induced damage (40). These effects involve cellular stress responses, autophagic adaptation, and multiple forms of programmed cell death. Among the microbial taxa implicated in these processes, *Fusobacterium nucleatum* (Fn) is one of the best-characterized bacteria in studies of microbiota-associated chemotherapy resistance in CRC (41).

Mechanistic studies have shown that Fn infection activates the TLR4/MyD88 signaling pathway and, through modulation of miR-18a* and miR-4802 expression, relieves their inhibitory effects on the autophagy-related proteins ULK1 and ATG7, thereby promoting protective autophagy (26). Pharmacological or genetic blockade of TLR4/MyD88 signaling or autophagy restores the sensitivity of CRC cells to 5-FU and OXA, suggesting that Fn enhances cellular stress adaptation and limits the accumulation of chemotherapy-induced damage. Beyond autophagy, Fn can also modulate other forms of programmed cell death. Wang et al. (27) reported that Fn promotes BCL2 expression through regulation of the Hippo/YAP signaling pathway, thereby suppressing chemotherapy-induced pyroptosis mediated by the Caspase-3/GSDME axis and increasing chemotherapy resistance in CRC cells. In addition, Fn can activate the E-cadherin/β-catenin/TCF4/GPX4 signaling axis, suppress ferroptosis, and promote OXA resistance (42). Collectively, these findings indicate that Fn may not act through a single resistance pathway but rather promote tumor cell survival under chemotherapy pressure by reshaping cellular responses to treatment-induced damage.

Beyond Fn, other functional microbial taxa and their metabolites may also contribute to the remodeling of tumor cell responses to chemotherapy-induced damage. Ueta et al. (43) reported that signals associated with *Citrobacter freundii* reduce NINJ2 expression, thereby limiting OXA-induced ROS accumulation and apoptosis and promoting OXA tolerance in CRC cells. These findings indicate that even low-abundance functional taxa may influence host damage responses through specific molecular pathways. More recently, trans-3-indoleacrylic acid, a metabolite derived from the CRC-enriched bacterium *Peptostreptococcus anaerobius*, was shown to activate the AhR–ALDH1A3 axis, enhance ferroptosis resistance, and promote tumor cell survival (44). Together, these findings support a model in which distinct microbial signals can converge on cellular stress adaptation and regulated cell death pathways, thereby contributing to tumor cell survival under chemotherapy pressure.

#### 3.2.2 Microbiota-induced remodeling of tumor cell plasticity and maintenance of cancer stemness

Enhanced tumor cell plasticity is an important basis for therapeutic adaptation in CRC. Epithelial–mesenchymal transition (EMT) and cancer stemness are closely interconnected and jointly drive tumor cell state remodeling, thereby promoting the acquisition of resistant phenotypes (45, 46). Enterotoxigenic *Bacteroides fragilis* (ETBF) secretes *B. fragilis* toxin (BFT), which cleaves E-cadherin, disrupts intercellular adhesion, and activates signaling pathways including Wnt/β-catenin, STAT3, and NF-κB, thereby inducing EMT (47–49). EMT is frequently accompanied by enhanced cancer stemness, resistance to apoptosis, and reduced drug sensitivity, suggesting that ETBF may contribute to chemotherapy resistance in CRC by promoting tumor cell state remodeling (50).

Ding et al. (28) reported that *B. fragilis* was enriched in patients with CRC who had poor chemotherapy responses and was associated with unfavorable clinical outcomes. Mechanistic studies further showed that the surface proteins SusD/RagB bind to the Notch1 receptor on CRC cells, activate Notch1 signaling, promote EMT and cancer stemness, and suppress apoptosis induced by 5-FU and OXA. Deletion of SusD/RagB, blockade of Notch1 signaling, or selective depletion of *B. fragilis* using the targeted phage VA7 restored chemotherapy sensitivity in CRC models (28), providing a rationale for precision targeting of resistance-associated microbial taxa.

Beyond *B. fragilis*, other oncogenic gut bacteria may also contribute to CRC chemotherapy resistance by promoting tumor cell plasticity. Dalmasso et al. (51) reported that colibactin-producing *Escherichia coli* can induce EMT and promote stem cell-like features, thereby increasing the tolerance of CRC cells to chemotherapeutic agents. Although these microbial taxa differ in their molecular effectors and signaling pathways, their effects may converge on tumor cell state remodeling, providing a common route toward adaptation to sustained therapeutic pressure.

#### 3.2.3 Microbiota-induced remodeling of the DNA damage response and tumor adaptation

Accumulation of DNA damage is a key mechanism underlying tumor cell death induced by 5-FU and OXA (52, 53). Sustained therapeutic pressure may reshape DNA damage responses associated with microbial exposure.

*Escherichia coli* carrying the pks genomic island (pks^+^ *E. coli*) produces the genotoxin colibactin, which induces DNA double-strand breaks and interstrand crosslinks and generates characteristic mutational signatures, thereby contributing to genomic instability and clonal evolution in CRC (30). Emerging evidence suggests that colibactin-induced alterations in DNA damage responses may influence tumor adaptation. Sogari et al. (29) reported that the ability of CRC cells to tolerate colibactin-induced damage was closely associated with homologous recombination repair capacity. Prolonged exposure to colibactin promoted the selective enrichment of tumor cell subpopulations with enhanced DNA repair capacity and was accompanied by increased tolerance to SN38. In addition, a retrospective analysis of patients with metastatic CRC treated with FOLFIRI showed a trend toward poorer treatment response in tumors harboring pks^+^ strains, suggesting that differences in colibactin-associated DNA damage tolerance and repair capacity may contribute to tumor adaptation under chemotherapy-induced selective pressure (29).

The gut microbiota may also influence tumor adaptation to chemotherapy by modulating DNA damage responses and redox homeostasis (43). However, the effects of microbiota-derived genotoxic factors such as colibactin are context dependent and may vary according to microbial exposure, the DNA repair landscape of the tumor, and the therapeutic regimen (29). Thus, microbiota-mediated alterations in cellular damage responses may function as evolutionary pressures that shape tumor adaptation and clonal selection rather than as independent determinants of chemotherapy resistance.

### 3.3 Microbiota-mediated remodeling of the immune microenvironment and post-treatment immune escape

The effects of the gut microbiota on chemotherapy responses in CRC extend beyond tumor cell-intrinsic mechanisms and are closely linked to chemotherapy-induced host immune responses. Certain chemotherapeutic agents can induce immunogenic cell death (ICD), promoting antigen presentation and T-cell activation through the release of ATP and high-mobility group box 1 (HMGB1) and the exposure of calreticulin, thereby enhancing antitumor immunity (54). Gut dysbiosis may alter these chemotherapy-induced immune responses, thereby weakening immune surveillance of residual tumor cells after treatment (55, 56).

Alterations in the composition and functional activity of the gut microbiota can influence the immune microenvironment after chemotherapy. In the context of innate immunity, microbial signals and metabolites can influence the function of dendritic cells and other myeloid cells, thereby affecting inflammatory responses and antigen presentation (57). For adaptive immunity, microbial metabolites can influence T-cell function and antitumor immune responses (58). CRC-associated dysbiosis has been linked to an immunosuppressive microenvironment and impaired immune surveillance (59). For example, beyond promoting tumor cell-intrinsic resistance, Fn can suppress antitumor immunity through its surface adhesin Fap2, which binds to the inhibitory receptor TIGIT on NK cells and T cells, thereby attenuating immune cell cytotoxicity and reducing the production of antitumor effector molecules such as IFN-γ. This immune-suppressive mechanism may further compromise the clearance of residual tumor cells following chemotherapy (60).

Collectively, microbiota-mediated immune regulation can shape the tumor immune microenvironment during chemotherapy and influence the survival and adaptation of residual tumor cells under therapeutic pressure. However, systematic evidence remains limited regarding how specific functional microbial taxa regulate chemotherapy-induced antitumor immune responses and whether these interactions directly contribute to the evolution of chemotherapy resistance and affect clinical outcomes in CRC.

### 3.4 Microbial metabolite-mediated regulation of chemotherapy tolerance

Microbiota-derived metabolites are important functional mediators through which the gut microbiota influences host responses to therapy (61, 62). They can affect therapeutic efficacy and toxicity by maintaining intestinal barrier homeostasis and modulating immune responses and drug metabolism (63). During chemotherapy, remodeling of microbial metabolic networks can further influence tumor cell adaptation and host antitumor immunity, thereby modulating chemotherapy sensitivity and treatment tolerance (64).

Butyrate is one of the most extensively studied short-chain fatty acids (SCFAs) and can influence the response of CRC cells to chemotherapy through mechanisms including histone deacetylase (HDAC) inhibition and modulation of immune responses (65). Studies have shown that gut microbiota depletion can impair the antitumor immune response induced by OXA, whereas microbiota-derived butyrate can enhance OXA efficacy by promoting IL-12 signaling and increasing the cytotoxic function of ID2-dependent CD8^+^ T cells (58). Clinical studies have further supported an association between butyrate-producing bacteria and treatment response. Li et al. (66) reported that dynamic changes in *Roseburia faecis* during chemotherapy were associated with treatment response, whereas Yi et al. (67) found that *Roseburia*, *Dorea*, and *Anaerostipes*, including butyrate-producing taxa, were enriched in the gut microbiota of patients who responded to neoadjuvant chemoradiotherapy before treatment. Functional validation by Gomes et al. (68) showed that a mixture of SCFAs combined with low-dose 5-FU enhanced the response to chemotherapy in CRC cells and animal models, supporting the potential role of microbiota-derived metabolites in modulating chemotherapy sensitivity.

Beyond SCFAs, microbiota-derived metabolites and metabolic functions, including secondary bile acids, tryptophan metabolites, and microbial nucleotide synthesis, have also been implicated in the regulation of cancer treatment responses (69, 70). Teng et al. (34) reported that *Bacteroides vulgatus*-mediated nucleotide biosynthesis was associated with reduced response to neoadjuvant chemoradiotherapy in patients with rectal cancer. Functional experiments further showed that nucleotide supplementation or *B. vulgatus* administration reduced the sensitivity of rectal cancer cells to 5-FU or irradiation, suggesting that microbial nucleotide biosynthesis may contribute to reduced treatment sensitivity by altering nucleotide availability and tumor cell metabolism. Microbiota-derived metabolites can modulate inflammation, immune cell function, and the tumor microenvironment through signaling pathways involving AhR, FXR, and TGR5 (71, 72). However, evidence that specific microbial metabolites or metabolic functions directly drive resistance to 5-FU or OXA remains limited, and current studies have focused primarily on their associations with treatment sensitivity and host responses.

Notably, luminal SCFAs primarily reflect the overall metabolic activity of the gut microbiota and mucosal ecological homeostasis (73), whereas the metabolic milieu within the tumor may have a more direct influence on tumor cell adaptation to chemotherapy-induced stress (74). Therefore, fecal or circulating metabolite levels alone may not fully capture the metabolic state of the tumor microenvironment. Future studies should characterize the spatial distribution of key microbiota-derived metabolites and determine how their localization relates to chemotherapy sensitivity (75, 76).

### 3.5 Network-level and cooperative mechanisms underlying microbiota-mediated chemotherapy resistance in CRC

Current evidence indicates that microbiota-mediated chemotherapy resistance in CRC involves multilayered interactions among microbial functional traits, host responses, and tumor cell adaptation rather than being attributable to a single microbial taxon (8, 55). Although distinct microbial taxa act through different molecular targets and mechanisms, their biological effects may converge on common outcomes, including sustained inflammatory activation, immunosuppression, enhanced tumor cell plasticity, and metabolic adaptation, reflecting functional convergence across microbial activities (9). Collectively, the available evidence suggests that CRC chemotherapy resistance may emerge from the coordinated remodeling of multiple resistance-associated functional modules under sustained therapeutic pressure, rather than from a single pathogenic bacterium or molecular mechanism.

From this perspective, interventions targeting a single microbial taxon or molecular target may be limited by functional redundancy within the microbial community and compensatory interactions within the ecological network (77, 78). Future studies should integrate longitudinal cohorts, multi-omics profiling, and host–microbiota interaction network analyses to identify key functional modules associated with heterogeneity in chemotherapy response and to develop precision microbiome-based interventions guided by functional regulation. Such approaches may provide a framework for patient stratification and the clinical translation of microbiome-based strategies for CRC chemotherapy resistance.

## 4 Microecological modulation and precision intervention strategies

### 4.1 Strategies for restoring microecological homeostasis and modulating microbial functions

Dietary interventions, probiotics, and prebiotics are among the most widely used strategies for modulating the gut microbiota (79). These approaches primarily aim to restore microbial homeostasis, improve intestinal barrier function, and regulate host inflammatory and immune responses, thereby mitigating chemotherapy-associated ecological disturbances (57). Multiple systematic reviews and meta-analyses have shown that probiotic or synbiotic supplementation can alleviate chemotherapy-related toxicities and partially ameliorate chemotherapy-associated gut dysbiosis (80–82). In addition, clinical studies suggest that probiotic interventions may partially restore chemotherapy-induced alterations in the gut microbiota and potentially improve treatment tolerability and selected clinical outcomes, providing preliminary support for microbiome modulation as an adjunctive strategy during chemotherapy (22).

In recent years, microecological intervention strategies have increasingly shifted from modifying microbial abundance toward modulating microbial functional states (83, 84). However, microbial functional outputs are highly context dependent, and their biological effects are shaped by the source of the producing taxa, the site of action, and the local tumor microenvironment (85). For example, although butyrate generally exerts immunomodulatory and barrier-protective effects, microbiota-derived butyrate may, under specific CRC microenvironmental conditions, activate PI3K–AKT-related signaling, promote adaptive tumor cell growth, and potentially reduce chemotherapy sensitivity (86). Therefore, microecological intervention should not be equated simply with increasing the abundance of “beneficial” taxa or metabolites; rather, interventions should be evaluated in the context of microbial functional origin, spatial distribution, and host background.

Most current studies use chemotherapy-associated diarrhea, mucositis, or changes in microbial diversity as primary endpoints, whereas evidence for improving tumor treatment response or reversing established chemotherapy resistance remains limited (81). In addition, probiotic strains differ in their colonization capacity and functional properties, contributing to heterogeneity across studies (87). Overall, current approaches to restoring microecological homeostasis primarily serve as adjunctive strategies to mitigate chemotherapy-associated ecological disturbances and improve treatment tolerability, whereas precise remodeling of resistance-associated functional modules remains an emerging strategy requiring further mechanistic and clinical validation (88).

### 4.2 Fecal microbiota transplantation

Fecal microbiota transplantation (FMT) is a whole-community microbiome intervention that involves transferring the gut microbiota from a donor to a recipient to reconstitute the recipient’s intestinal microbial ecosystem (89). Studies of FMT in patients with melanoma who developed resistance to immune checkpoint inhibitors have suggested that microbiome remodeling can influence antitumor treatment responses, providing evidence for the potential role of microbiome modulation in treatment response (90, 91). However, these findings were obtained in melanoma and immunotherapy settings and should not be directly extrapolated to chemotherapy resistance in CRC, given differences in tumor type, treatment modality, and host–microbiota interactions.

To date, studies investigating FMT for chemotherapy resistance in CRC remain largely at the preclinical stage. Chang et al. (92) reported that FMT alleviated chemotherapy-induced gut dysbiosis and reduced 5-FU- and OXA-associated intestinal mucosal injury in CRC models. Further studies using patient-derived xenograft models of CRC showed that FMT altered recipient gut microbial composition and enhanced FOLFOX efficacy in selected models, suggesting that FMT may influence treatment sensitivity by restoring microbiome states associated with chemotherapy responsiveness (93). However, most studies have focused on mitigating treatment-related gastrointestinal toxicity, whereas clinical evidence for improved tumor response or reversal of chemotherapy resistance remains limited (94, 95). As a complex live microbial community intervention, FMT efficacy may depend on multiple factors, including donor microbial functional profiles, the recipient’s baseline microbiome, and post-transplant engraftment capacity. Variations in chemotherapy regimens, FMT protocols, and response assessment criteria across studies further complicate the evaluation of its clinical utility (96).

Thus, the future development of FMT should focus not simply on restoring a “healthy microbiota,” but on identifying functional ecological states associated with treatment response. Future studies should integrate donor microbial functional characteristics, recipient baseline microbiome profiles, engraftment dynamics, and treatment response data to identify the microbial functional modules and host response features that predict FMT benefit. Such an approach could advance FMT from whole-community microbiome modulation toward predictable, function-guided, and precision-guided microbiome intervention.

### 4.3 Precision targeting of resistance-associated microbes and functional effectors

Compared with whole-community microbiome remodeling, precision interventions targeting resistance-promoting microbes and their key functional effectors may offer a more direct approach to restoring chemotherapy sensitivity (97). Ding et al. (28) reported that *Bacteroides fragilis* (*B. fragilis*) was enriched in patients with CRC who had poor responses to chemotherapy and that its abundance was associated with poorer survival. In cellular and murine models, the bacterial surface proteins SusD/RagB were shown to bind the Notch1 receptor and activate Notch1 signaling, thereby promoting EMT and cancer stemness and reducing the sensitivity of CRC cells to 5-FU and OXA. Selective depletion of *B. fragilis* using the bacterium-specific phage VA7 reduced bacterial burden and restored the response to chemotherapy in experimental models, highlighting the potential translational value of targeting resistance-associated microbial taxa.

Precision modulation of microbial functional outputs represents another important area of investigation (33). Bacterial β-glucuronidase can hydrolyze the inactive conjugate SN-38G to the active metabolite SN-38, increasing luminal exposure to SN-38 and exacerbating gastrointestinal toxicity (32). Small-molecule inhibitors of bacterial GUS can block SN-38 reactivation and attenuate irinotecan-associated intestinal injury without substantially altering the overall structure of the gut microbiota (39). Although this strategy has primarily been developed to mitigate chemotherapy-related toxicity, it demonstrates the feasibility of selectively modulating specific microbial functions while preserving the broader microbial community structure, providing an important proof of concept for targeting microbial functional nodes without broadly disrupting the microbiome. However, the clinical translation of this approach remains challenging. Gut microbial GUS enzymes exhibit substantial structural and functional diversity, which may affect the selectivity and efficacy of inhibitors across different microbial GUS enzymes. Moreover, current evidence is predominantly preclinical, and the safety, pharmacological properties, and clinical efficacy of GUS inhibitors require further validation in humans (37).

The effects of precision microbiome interventions may depend on the microbial ecological context and host factors. For resistance-associated taxa such as *B. fragilis*, depletion of a single taxon may not produce consistent therapeutic effects, particularly when other microorganisms provide overlapping or compensatory functions. Therefore, treatment efficacy should be evaluated not only by changes in microbial abundance but also by functional changes and their association with chemotherapy response. Selective targeting of specific microbial taxa or functional pathways may also affect microbial interactions and ecological stability. Importantly, evidence that precision microbiome interventions can reverse established chemotherapy resistance in CRC remains largely preclinical, and their clinical efficacy, durability, and safety remain to be established (28, 98). Future studies should define microbial functions consistently associated with chemotherapy response and evaluate their therapeutic potential across different ecological and host contexts.

## 5 Clinical translation challenges and future directions

The gut microbiome is characterized by substantial interindividual heterogeneity and dynamic variation. Factors such as age, diet, antibiotic exposure, and chemotherapy regimen can influence microbial composition and function (33), complicating the identification of resistance-associated features and the development of microbiome-based biomarkers. Moreover, inconsistencies in sample collection procedures, sequencing technologies, definitions of chemotherapy resistance, and criteria for treatment response assessment across studies limit reproducibility and clinical generalizability (99). Notably, differences in tumor biology, treatment modality, and host–microbiota interactions across cancer types may result in distinct effects of the microbiome on treatment response. Therefore, mechanisms identified in other cancer types require further validation in CRC-specific chemotherapy settings to clarify their clinical relevance to chemotherapy resistance in CRC.

From an interventional perspective, whole-community microbiome modulation strategies, including FMT and probiotics, show potential, but their mechanisms of action, treatment consistency, and optimal patient populations remain to be established (83, 100). Precision interventions targeting resistance-associated microbial taxa or specific microbial functions must not only improve therapeutic selectivity but also be evaluated systematically for their effects on the broader ecological network and long-term microbiome stability. Antibiotic exposure represents an additional clinical factor that may influence this process. Antibiotics can alter gut microbial composition and impose selective pressure on microbial communities, potentially affecting microbial functions and subsequent microbiome modulation (101). Antibiotic exposure may also favor the enrichment of antibiotic-resistant organisms, further altering the functional properties of the gut microbiome. These factors should therefore be considered when evaluating the efficacy and clinical applicability of microbiome-based interventions.

Future research should further bridge mechanistic investigation and clinical translation. At the mechanistic level, metagenomics, metabolomics, spatial multi-omics, and functional models could be integrated to identify potential causal relationships between microbial functions, their metabolites, and host resistance pathways, followed by targeted modulation of relevant microorganisms or microbial activities to validate candidate functional features. At the translational level, microbial functional features, microbiota-derived metabolites, and relevant host characteristics could serve as candidate components of biomarker frameworks for patient stratification to identify patients who are more likely to benefit from microbiome-based interventions. These candidate biomarkers will require further validation in independent patient cohorts.

## 6 Conclusion

Current evidence indicates that the gut microbiome can influence chemotherapy response in colorectal cancer through multiple interconnected mechanisms involving drug metabolism and exposure, tumor cell adaptation, immune regulation, and microbiota-derived metabolites. Among these mechanisms, microbial modulation of drug metabolism and the functional roles of resistance-associated taxa, particularly *Fusobacterium nucleatum* and *Bacteroides fragilis*, are supported by relatively stronger evidence, whereas the contributions of other pathways remain less clearly defined. Overall, chemotherapy resistance appears to involve the interaction and convergence of multiple microbial and host processes rather than a single microbial taxon or pathway. Future studies should prioritize reproducible microbial functional features, followed by functional validation of their causal roles and evaluation of their potential as biomarkers for patient stratification. This stepwise approach may provide a stronger foundation for precision microbiome-based strategies for CRC chemotherapy.
